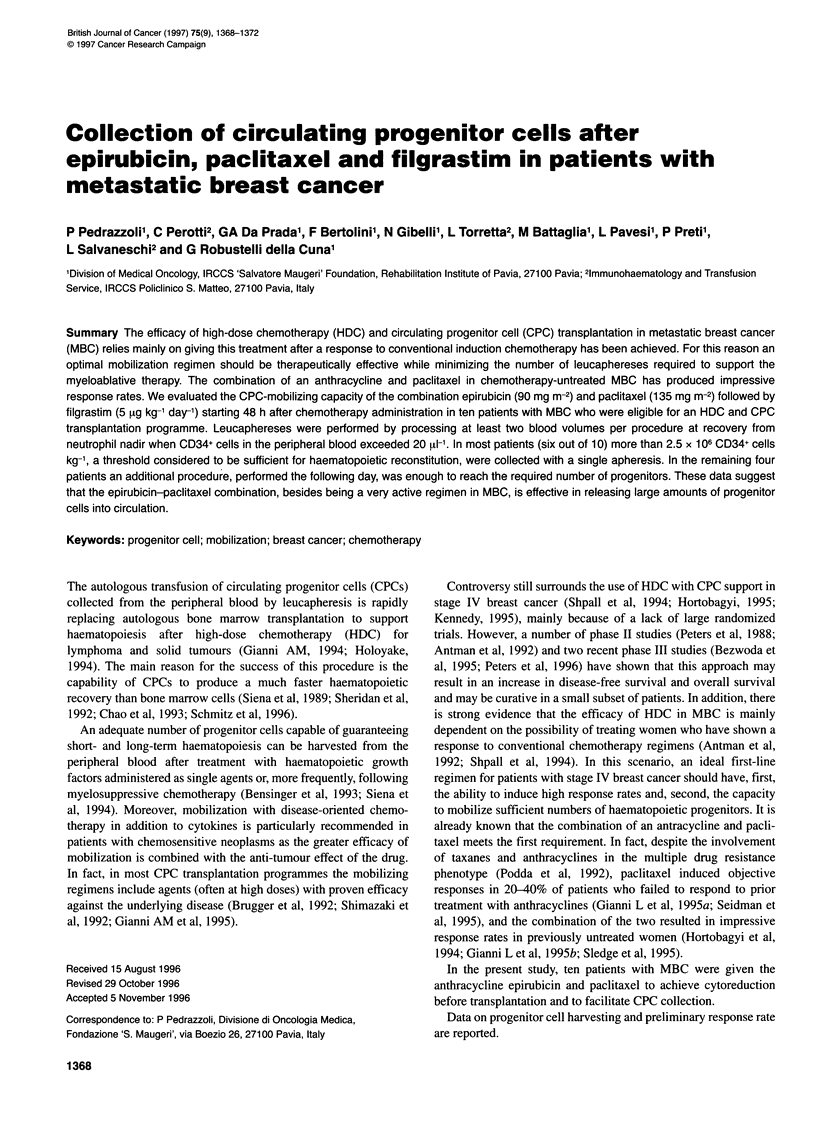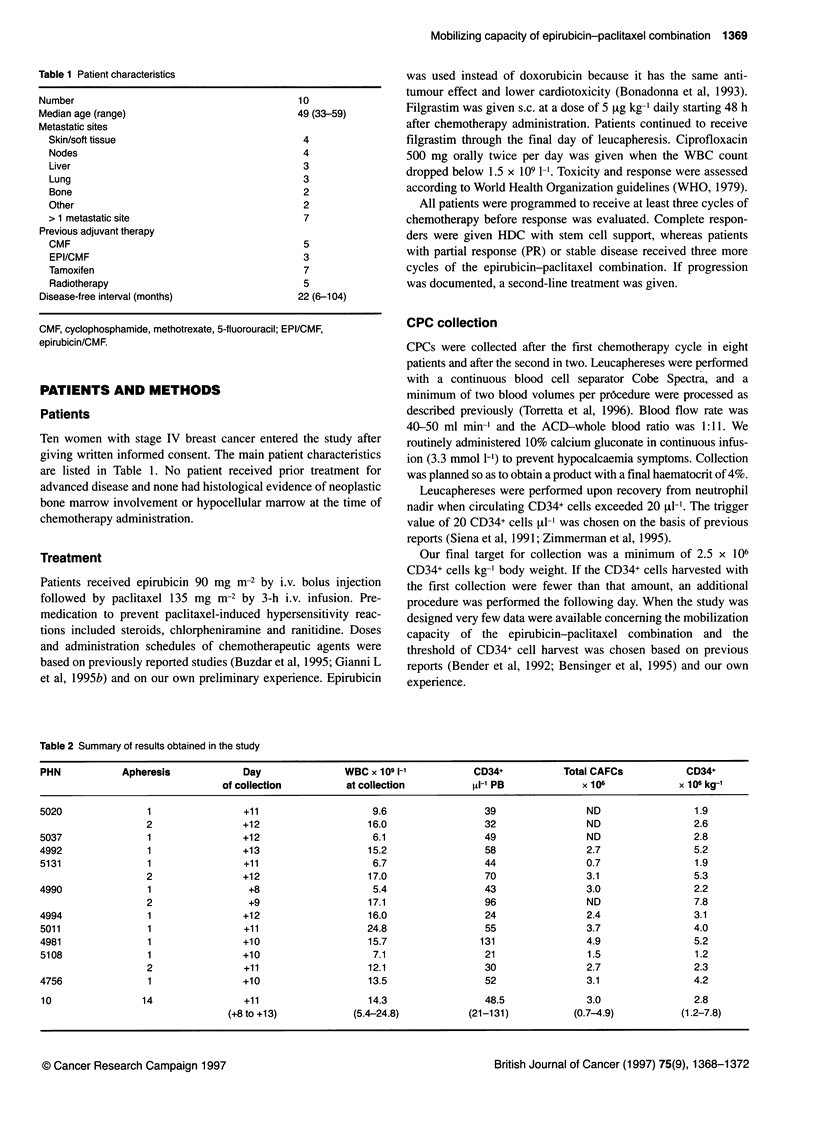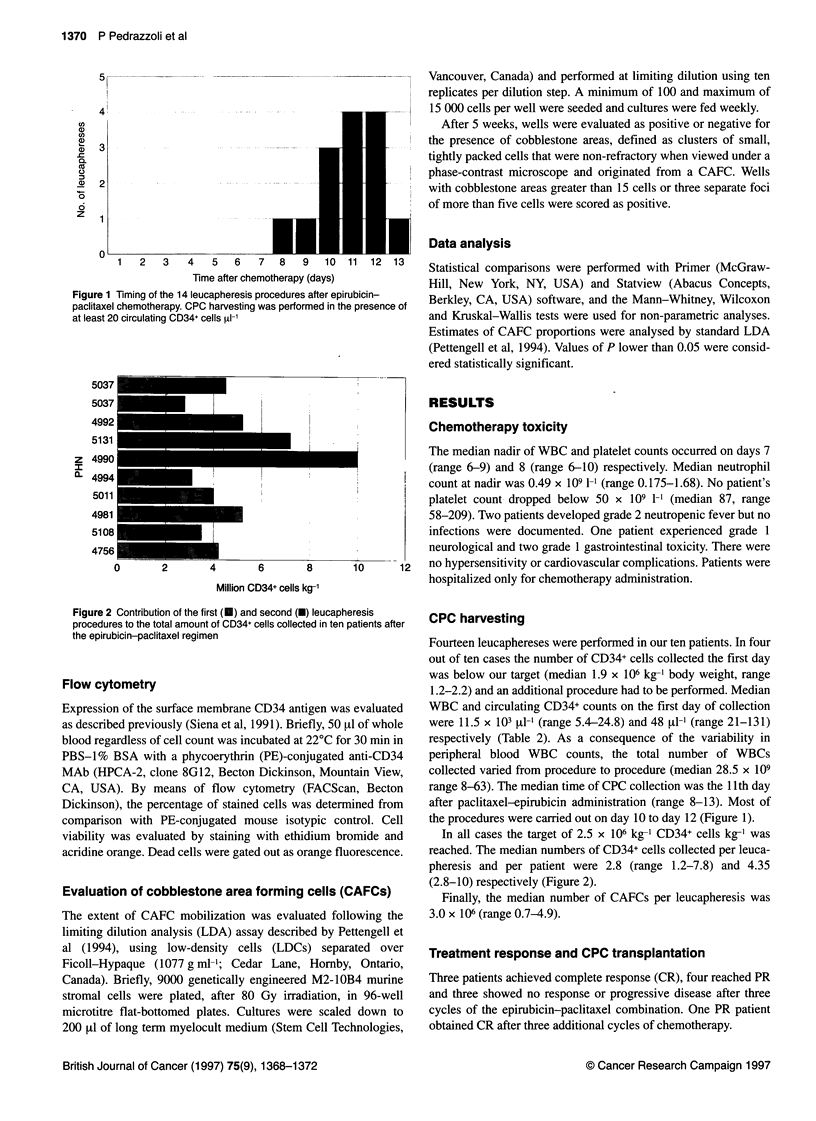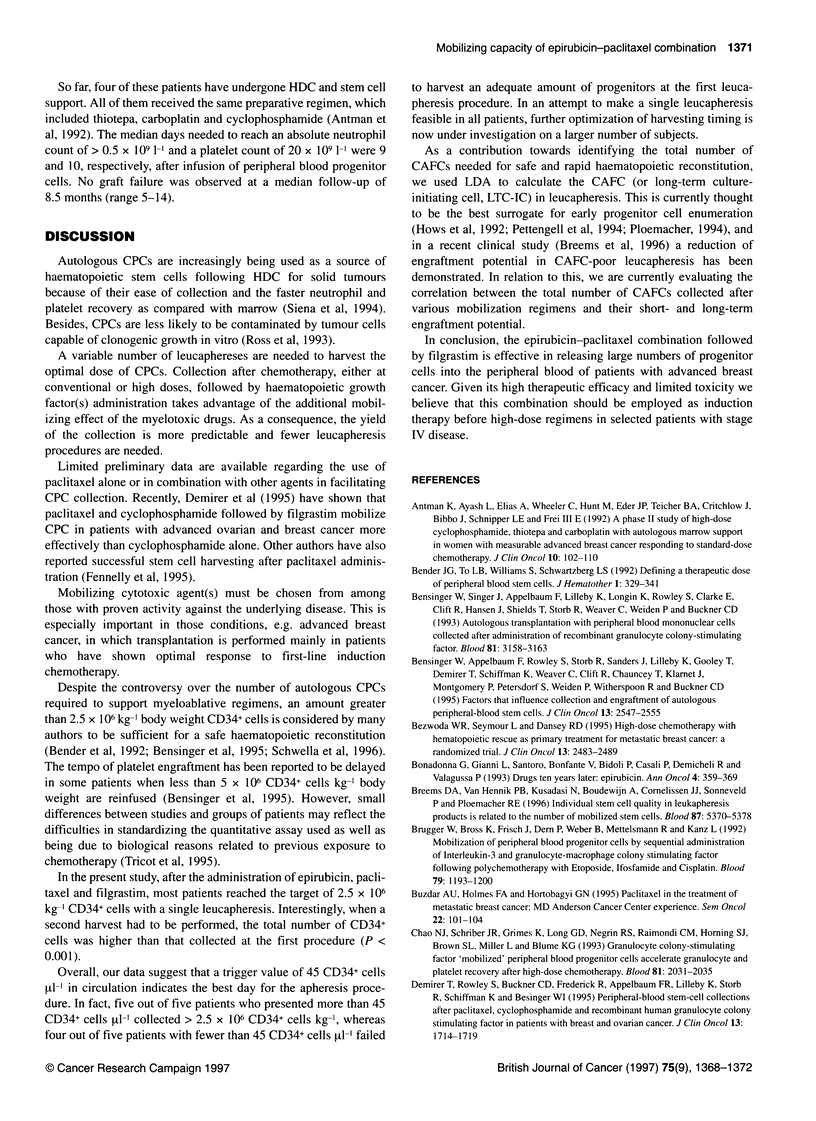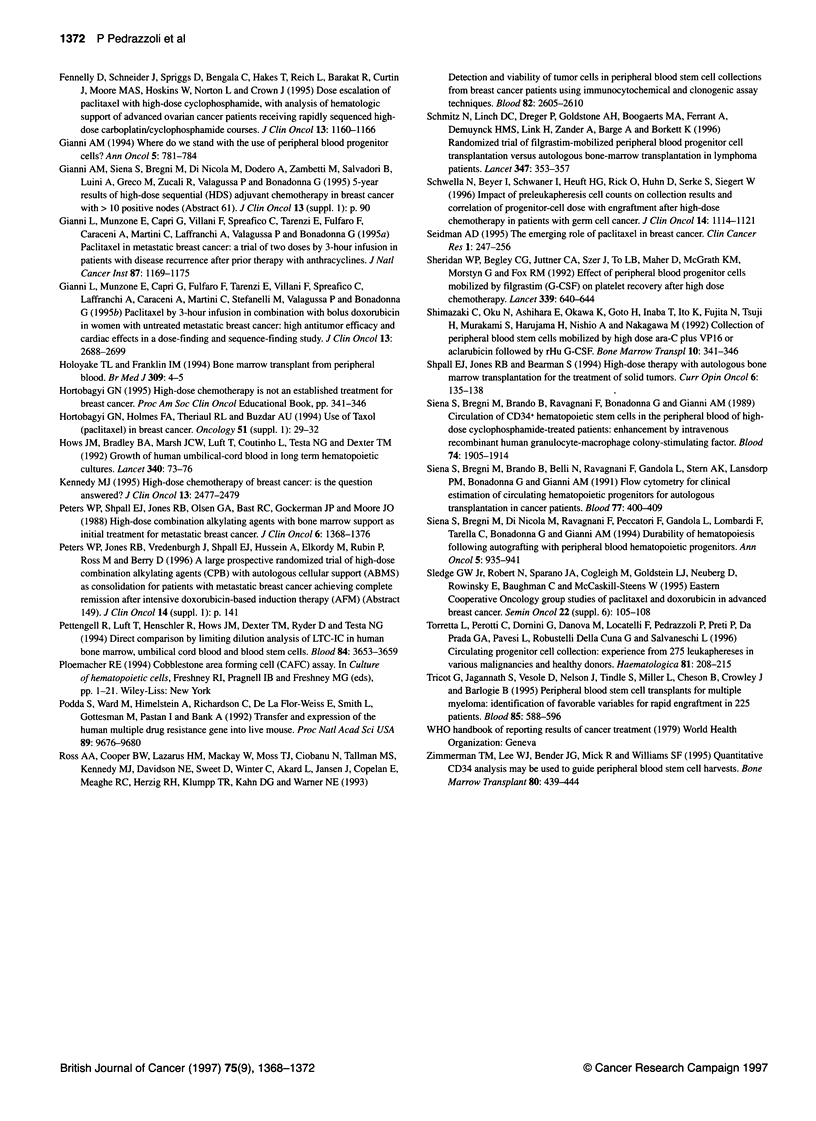# Collection of circulating progenitor cells after epirubicin, paclitaxel and filgrastim in patients with metastatic breast cancer.

**DOI:** 10.1038/bjc.1997.231

**Published:** 1997

**Authors:** P. Pedrazzoli, C. Perotti, G. A. Da Prada, F. Bertolini, N. Gibelli, L. Torretta, M. Battaglia, L. Pavesi, P. Preti, L. Salvaneschi, G. Robustelli della Cuna

**Affiliations:** Division of Medical Oncology, IRCCS Salvatore Maugeri Foundation, Rehabilitation Institute of Pavia, Italy.

## Abstract

The efficacy of high-dose chemotherapy (HDC) and circulating progenitor cell (CPC) transplantation in metastatic breast cancer (MBC) relies mainly on giving this treatment after a response to conventional induction chemotherapy has been achieved. For this reason an optimal mobilization regimen should be therapeutically effective while minimizing the number of leucaphereses required to support the myeloablative therapy. The combination of an anthracycline and paclitaxel in chemotherapy-untreated MBC has produced impressive response rates. We evaluated the CPC-mobilizing capacity of the combination epirubicin (90 mg m(-2)) and paclitaxel (135 mg m(-2)) followed by filgrastim (5 microg kg(-1) day(-1)) starting 48 h after chemotherapy administration in ten patients with MBC who were eligible for an HDC and CPC transplantation programme. Leucaphereses were performed by processing at least two blood volumes per procedure at recovery from neutrophil nadir when CD34+ cells in the peripheral blood exceeded 20 microl(-1). In most patients (six out of 10) more than 2.5 x 10(6) CD34+ cells kg(-1), a threshold considered to be sufficient for haematopoietic reconstitution, were collected with a single apheresis. In the remaining four patients an additional procedure, performed the following day, was enough to reach the required number of progenitors. These data suggest that the epirubicin-paclitaxel combination, besides being a very active regimen in MBC, is effective in releasing large amounts of progenitor cells into circulation.


British Journal of Cancer (1997) 75(9), 1368–1372
© 1997 Cancer Research Campaign

### Collection of circulating progenitor cells after epirubicin, paclitaxel and filgrastim in patients with metastatic breast cancer

**P Pedrazzoli[1], C Perotti[2], GA Da Prada[1], F Bertolini[1], N Gibelli[1], L Torretta[2], M Battaglia[1], L Pavesi[1], P Preti[1], L Salvaneschi[2] and G Robustelli della Cuna[1]**

[1]Division of Medical Oncology, IRCCS 'Salvatore Maugeri' Foundation, Rehabilitation Institute of Pavia, 27100 Pavia; [2]Immunohaematology and Transfusion Service, IRCCS Policlinico S. Matteo, 27100 Pavia, Italy

**Summary** The efficacy of high-dose chemotherapy (HDC) and circulating progenitor cell (CPC) transplantation in metastatic breast cancer (MBC) relies mainly on giving this treatment after a response to conventional induction chemotherapy has been achieved. For this reason an optimal mobilization regimen should be therapeutically effective while minimizing the number of leucaphereses required to support the myeloablative therapy. The combination of an anthracycline and paclitaxel in chemotherapy-untreated MBC has produced impressive response rates. We evaluated the CPC-mobilizing capacity of the combination epirubicin (90 mg $m^{-2}$) and paclitaxel (135 mg $m^{-2}$) followed by filgrastim (5 $\mu$g $kg^{-1}$ $day^{-1}$) starting 48 h after chemotherapy administration in ten patients with MBC who were eligible for an HDC and CPC transplantation programme. Leucaphereses were performed by processing at least two blood volumes per procedure at recovery from neutrophil nadir when $CD34^+$ cells in the peripheral blood exceeded 20 $\mu l^{-1}$. In most patients (six out of 10) more than $2.5 \times 10^6$ $CD34^+$ cells $kg^{-1}$, a threshold considered to be sufficient for haematopoietic reconstitution, were collected with a single apheresis. In the remaining four patients an additional procedure, performed the following day, was enough to reach the required number of progenitors. These data suggest that the epirubicin–paclitaxel combination, besides being a very active regimen in MBC, is effective in releasing large amounts of progenitor cells into circulation.

**Keywords:** progenitor cell; mobilization; breast cancer; chemotherapy

The autologous transfusion of circulating progenitor cells (CPCs) collected from the peripheral blood by leucapheresis is rapidly replacing autologous bone marrow transplantation to support haematopoiesis after high-dose chemotherapy (HDC) for lymphoma and solid tumours (Gianni AM, 1994; Holoyake, 1994). The main reason for the success of this procedure is the capability of CPCs to produce a much faster haematopoietic recovery than bone marrow cells (Siena et al, 1989; Sheridan et al, 1992; Chao et al, 1993; Schmitz et al, 1996).

An adequate number of progenitor cells capable of guaranteeing short- and long-term haematopoiesis can be harvested from the peripheral blood after treatment with haematopoietic growth factors administered as single agents or, more frequently, following myelosuppressive chemotherapy (Bensinger et al, 1993; Siena et al, 1994). Moreover, mobilization with disease-oriented chemotherapy in addition to cytokines is particularly recommended in patients with chemosensitive neoplasms as the greater efficacy of mobilization is combined with the anti-tumour effect of the drug. In fact, in most CPC transplantation programmes the mobilizing regimens include agents (often at high doses) with proven efficacy against the underlying disease (Brugger et al, 1992; Shimazaki et al, 1992; Gianni AM et al, 1995).

Received 15 August 1996
Revised 29 October 1996
Accepted 5 November 1996

Correspondence to: P Pedrazzoli, Divisione di Oncologia Medica, Fondazione 'S. Maugeri', via Boezio 26, 27100 Pavia, Italy

Controversy still surrounds the use of HDC with CPC support in stage IV breast cancer (Shpall et al, 1994; Hortobagyi, 1995; Kennedy, 1995), mainly because of a lack of large randomized trials. However, a number of phase II studies (Peters et al, 1988; Antman et al, 1992) and two recent phase III studies (Bezwoda et al, 1995; Peters et al, 1996) have shown that this approach may result in an increase in disease-free survival and overall survival and may be curative in a small subset of patients. In addition, there is strong evidence that the efficacy of HDC in MBC is mainly dependent on the possibility of treating women who have shown a response to conventional chemotherapy regimens (Antman et al, 1992; Shpall et al, 1994). In this scenario, an ideal first-line regimen for patients with stage IV breast cancer should have, first, the ability to induce high response rates and, second, the capacity to mobilize sufficient numbers of haematopoietic progenitors. It is already known that the combination of an antracycline and paclitaxel meets the first requirement. In fact, despite the involvement of taxanes and anthracyclines in the multiple drug resistance phenotype (Podda et al, 1992), paclitaxel induced objective responses in 20–40% of patients who failed to respond to prior treatment with anthracyclines (Gianni L et al, 1995*a*; Seidman et al, 1995), and the combination of the two resulted in impressive response rates in previously untreated women (Hortobagyi et al, 1994; Gianni L et al, 1995*b*; Sledge et al, 1995).

In the present study, ten patients with MBC were given the anthracycline epirubicin and paclitaxel to achieve cytoreduction before transplantation and to facilitate CPC collection.

Data on progenitor cell harvesting and preliminary response rate are reported.

**Table 1** Patient characteristics

| | |
|---|---|
| Number | 10 |
| Median age (range) | 49 (33–59) |
| Metastatic sites | |
| Skin/soft tissue | 4 |
| Nodes | 4 |
| Liver | 3 |
| Lung | 3 |
| Bone | 2 |
| Other | 2 |
| > 1 metastatic site | 7 |
| Previous adjuvant therapy | |
| CMF | 5 |
| EPI/CMF | 3 |
| Tamoxifen | 7 |
| Radiotherapy | 5 |
| Disease-free interval (months) | 22 (6–104) |

CMF, cyclophosphamide, methotrexate, 5-fluorouracil; EPI/CMF, epirubicin/CMF.

### PATIENTS AND METHODS

### Patients

Ten women with stage IV breast cancer entered the study after giving written informed consent. The main patient characteristics are listed in Table 1. No patient received prior treatment for advanced disease and none had histological evidence of neoplastic bone marrow involvement or hypocellular marrow at the time of chemotherapy administration.

### Treatment

Patients received epirubicin 90 mg $m^{-2}$ by i.v. bolus injection followed by paclitaxel 135 mg $m^{-2}$ by 3-h i.v. infusion. Premedication to prevent paclitaxel-induced hypersensitivity reactions included steroids, chlorpheniramine and ranitidine. Doses and administration schedules of chemotherapeutic agents were based on previously reported studies (Buzdar et al, 1995; Gianni L et al, 1995*b*) and on our own preliminary experience. Epirubicin was used instead of doxorubicin because it has the same antitumour effect and lower cardiotoxicity (Bonadonna et al, 1993). Filgrastim was given s.c. at a dose of 5 μg $kg^{-1}$ daily starting 48 h after chemotherapy administration. Patients continued to receive filgrastim through the final day of leucapheresis. Ciprofloxacin 500 mg orally twice per day was given when the WBC count dropped below $1.5 \times 10^9$ $l^{-1}$. Toxicity and response were assessed according to World Health Organization guidelines (WHO, 1979).

All patients were programmed to receive at least three cycles of chemotherapy before response was evaluated. Complete responders were given HDC with stem cell support, whereas patients with partial response (PR) or stable disease received three more cycles of the epirubicin–paclitaxel combination. If progression was documented, a second-line treatment was given.

### CPC collection

CPCs were collected after the first chemotherapy cycle in eight patients and after the second in two. Leucaphereses were performed with a continuous blood cell separator Cobe Spectra, and a minimum of two blood volumes per procedure were processed as described previously (Torretta et al, 1996). Blood flow rate was 40–50 ml $min^{-1}$ and the ACD–whole blood ratio was 1:11. We routinely administered 10% calcium gluconate in continuous infusion (3.3 mmol $l^{-1}$) to prevent hypocalcaemia symptoms. Collection was planned so as to obtain a product with a final haematocrit of 4%.

Leucaphereses were performed upon recovery from neutrophil nadir when circulating $CD34^+$ cells exceeded 20 $\mu l^{-1}$. The trigger value of 20 $CD34^+$ cells $\mu l^{-1}$ was chosen on the basis of previous reports (Siena et al, 1991; Zimmerman et al, 1995).

Our final target for collection was a minimum of $2.5 \times 10^6$ $CD34^+$ cells $kg^{-1}$ body weight. If the $CD34^+$ cells harvested with the first collection were fewer than that amount, an additional procedure was performed the following day. When the study was designed very few data were available concerning the mobilization capacity of the epirubicin–paclitaxel combination and the threshold of $CD34^+$ cell harvest was chosen based on previous reports (Bender et al, 1992; Bensinger et al, 1995) and our own experience.

**Table 2** Summary of results obtained in the study

| PHN | Apheresis | Day of collection | WBC $\times 10^9$ $l^{-1}$ at collection | $CD34^+$ $\mu l^{-1}$ PB | Total CAFCs $\times 10^6$ | $CD34^+$ $\times 10^6$ $kg^{-1}$ |
|---|---|---|---|---|---|---|
| 5020 | 1 | +11 | 9.6 | 39 | ND | 1.9 |
| | 2 | +12 | 16.0 | 32 | ND | 2.6 |
| 5037 | 1 | +12 | 6.1 | 49 | ND | 2.8 |
| 4992 | 1 | +13 | 15.2 | 58 | 2.7 | 5.2 |
| 5131 | 1 | +11 | 6.7 | 44 | 0.7 | 1.9 |
| | 2 | +12 | 17.0 | 70 | 3.1 | 5.3 |
| 4990 | 1 | +8 | 5.4 | 43 | 3.0 | 2.2 |
| | 2 | +9 | 17.1 | 96 | ND | 7.8 |
| 4994 | 1 | +12 | 16.0 | 24 | 2.4 | 3.1 |
| 5011 | 1 | +11 | 24.8 | 55 | 3.7 | 4.0 |
| 4981 | 1 | +10 | 15.7 | 131 | 4.9 | 5.2 |
| 5108 | 1 | +10 | 7.1 | 21 | 1.5 | 1.2 |
| | 2 | +11 | 12.1 | 30 | 2.7 | 2.3 |
| 4756 | 1 | +10 | 13.5 | 52 | 3.1 | 4.2 |
| 10 | 14 | +11 (+8 to +13) | 14.3 (5.4–24.8) | 48.5 (21–131) | 3.0 (0.7–4.9) | 2.8 (1.2–7.8) |

© Cancer Research Campaign 1997

**Figure 1** Timing of the 14 leucapheresis procedures after epirubicin–paclitaxel chemotherapy. CPC harvesting was performed in the presence of at least 20 circulating CD34+ cells μl$^{-1}$

**Figure 2** Contribution of the first (■) and second (■) leucapheresis procedures to the total amount of CD34+ cells collected in ten patients after the epirubicin–paclitaxel regimen

### Flow cytometry

Expression of the surface membrane CD34 antigen was evaluated as described previously (Siena et al, 1991). Briefly, 50 μl of whole blood regardless of cell count was incubated at 22°C for 30 min in PBS–1% BSA with a phycoerythrin (PE)-conjugated anti-CD34 MAb (HPCA-2, clone 8G12, Becton Dickinson, Mountain View, CA, USA). By means of flow cytometry (FACScan, Becton Dickinson), the percentage of stained cells was determined from comparison with PE-conjugated mouse isotypic control. Cell viability was evaluated by staining with ethidium bromide and acridine orange. Dead cells were gated out as orange fluorescence.

### Evaluation of cobblestone area forming cells (CAFCs)

The extent of CAFC mobilization was evaluated following the limiting dilution analysis (LDA) assay described by Pettengell et al (1994), using low-density cells (LDCs) separated over Ficoll–Hypaque (1077 g ml$^{-1}$; Cedar Lane, Hornby, Ontario, Canada). Briefly, 9000 genetically engineered M2-10B4 murine stromal cells were plated, after 80 Gy irradiation, in 96-well microtitre flat-bottomed plates. Cultures were scaled down to 200 μl of long term myelocult medium (Stem Cell Technologies, Vancouver, Canada) and performed at limiting dilution using ten replicates per dilution step. A minimum of 100 and maximum of 15 000 cells per well were seeded and cultures were fed weekly.

After 5 weeks, wells were evaluated as positive or negative for the presence of cobblestone areas, defined as clusters of small, tightly packed cells that were non-refractory when viewed under a phase-contrast microscope and originated from a CAFC. Wells with cobblestone areas greater than 15 cells or three separate foci of more than five cells were scored as positive.

### Data analysis

Statistical comparisons were performed with Primer (McGraw-Hill, New York, NY, USA) and Statview (Abacus Concepts, Berkley, CA, USA) software, and the Mann–Whitney, Wilcoxon and Kruskal–Wallis tests were used for non-parametric analyses. Estimates of CAFC proportions were analysed by standard LDA (Pettengell et al, 1994). Values of $P$ lower than 0.05 were considered statistically significant.

### RESULTS

### Chemotherapy toxicity

The median nadir of WBC and platelet counts occurred on days 7 (range 6–9) and 8 (range 6–10) respectively. Median neutrophil count at nadir was 0.49 × $10^9$ l$^{-1}$ (range 0.175–1.68). No patient's platelet count dropped below 50 × $10^9$ l$^{-1}$ (median 87, range 58–209). Two patients developed grade 2 neutropenic fever but no infections were documented. One patient experienced grade 1 neurological and two grade 1 gastrointestinal toxicity. There were no hypersensitivity or cardiovascular complications. Patients were hospitalized only for chemotherapy administration.

### CPC harvesting

Fourteen leucaphereses were performed in our ten patients. In four out of ten cases the number of CD34+ cells collected the first day was below our target (median 1.9 × $10^6$ kg$^{-1}$ body weight, range 1.2–2.2) and an additional procedure had to be performed. Median WBC and circulating CD34+ counts on the first day of collection were 11.5 × $10^3$ μl$^{-1}$ (range 5.4–24.8) and 48 μl$^{-1}$ (range 21–131) respectively (Table 2). As a consequence of the variability in peripheral blood WBC counts, the total number of WBCs collected varied from procedure to procedure (median 28.5 × $10^9$ range 8–63). The median time of CPC collection was the 11th day after paclitaxel–epirubicin administration (range 8–13). Most of the procedures were carried out on day 10 to day 12 (Figure 1).

In all cases the target of 2.5 × $10^6$ kg$^{-1}$ CD34+ cells kg$^{-1}$ was reached. The median numbers of CD34+ cells collected per leucapheresis and per patient were 2.8 (range 1.2–7.8) and 4.35 (2.8–10) respectively (Figure 2).

Finally, the median number of CAFCs per leucapheresis was 3.0 × $10^6$ (range 0.7–4.9).

### Treatment response and CPC transplantation

Three patients achieved complete response (CR), four reached PR and three showed no response or progressive disease after three cycles of the epirubicin–paclitaxel combination. One PR patient obtained CR after three additional cycles of chemotherapy.

© Cancer Research Campaign 1997

So far, four of these patients have undergone HDC and stem cell support. All of them received the same preparative regimen, which included thiotepa, carboplatin and cyclophosphamide (Antman et al, 1992). The median days needed to reach an absolute neutrophil count of > $0.5 \times 10^9$ $l^{-1}$ and a platelet count of $20 \times 10^9$ $l^{-1}$ were 9 and 10, respectively, after infusion of peripheral blood progenitor cells. No graft failure was observed at a median follow-up of 8.5 months (range 5–14).

### DISCUSSION

Autologous CPCs are increasingly being used as a source of haematopoietic stem cells following HDC for solid tumours because of their ease of collection and the faster neutrophil and platelet recovery as compared with marrow (Siena et al, 1994). Besides, CPCs are less likely to be contaminated by tumour cells capable of clonogenic growth in vitro (Ross et al, 1993).

A variable number of leucaphereses are needed to harvest the optimal dose of CPCs. Collection after chemotherapy, either at conventional or high doses, followed by haematopoietic growth factor(s) administration takes advantage of the additional mobilizing effect of the myelotoxic drugs. As a consequence, the yield of the collection is more predictable and fewer leucapheresis procedures are needed.

Limited preliminary data are available regarding the use of paclitaxel alone or in combination with other agents in facilitating CPC collection. Recently, Demirer et al (1995) have shown that paclitaxel and cyclophosphamide followed by filgrastim mobilize CPC in patients with advanced ovarian and breast cancer more effectively than cyclophosphamide alone. Other authors have also reported successful stem cell harvesting after paclitaxel administration (Fennelly et al, 1995).

Mobilizing cytotoxic agent(s) must be chosen from among those with proven activity against the underlying disease. This is especially important in those conditions, e.g. advanced breast cancer, in which transplantation is performed mainly in patients who have shown optimal response to first-line induction chemotherapy.

Despite the controversy over the number of autologous CPCs required to support myeloablative regimens, an amount greater than $2.5 \times 10^6$ $kg^{-1}$ body weight $CD34^+$ cells is considered by many authors to be sufficient for a safe haematopoietic reconstitution (Bender et al, 1992; Bensinger et al, 1995; Schwella et al, 1996). The tempo of platelet engraftment has been reported to be delayed in some patients when less than $5 \times 10^6$ $CD34^+$ cells $kg^{-1}$ body weight are reinfused (Bensinger et al, 1995). However, small differences between studies and groups of patients may reflect the difficulties in standardizing the quantitative assay used as well as being due to biological reasons related to previous exposure to chemotherapy (Tricot et al, 1995).

In the present study, after the administration of epirubicin, paclitaxel and filgrastim, most patients reached the target of $2.5 \times 10^6$ $kg^{-1}$ $CD34^+$ cells with a single leucapheresis. Interestingly, when a second harvest had to be performed, the total number of $CD34^+$ cells was higher than that collected at the first procedure ($P <$ 0.001).

Overall, our data suggest that a trigger value of 45 $CD34^+$ cells $\mu l^{-1}$ in circulation indicates the best day for the apheresis procedure. In fact, five out of five patients who presented more than 45 $CD34^+$ cells $\mu l^{-1}$ collected > $2.5 \times 10^6$ $CD34^+$ cells $kg^{-1}$, whereas four out of five patients with fewer than 45 $CD34^+$ cells $\mu l^{-1}$ failed to harvest an adequate amount of progenitors at the first leucapheresis procedure. In an attempt to make a single leucapheresis feasible in all patients, further optimization of harvesting timing is now under investigation on a larger number of subjects.

As a contribution towards identifying the total number of CAFCs needed for safe and rapid haematopoietic reconstitution, we used LDA to calculate the CAFC (or long-term culture-initiating cell, LTC-IC) in leucapheresis. This is currently thought to be the best surrogate for early progenitor cell enumeration (Hows et al, 1992; Pettengell et al, 1994; Ploemacher, 1994), and in a recent clinical study (Breems et al, 1996) a reduction of engraftment potential in CAFC-poor leucapheresis has been demonstrated. In relation to this, we are currently evaluating the correlation between the total number of CAFCs collected after various mobilization regimens and their short- and long-term engraftment potential.

In conclusion, the epirubicin–paclitaxel combination followed by filgrastim is effective in releasing large numbers of progenitor cells into the peripheral blood of patients with advanced breast cancer. Given its high therapeutic efficacy and limited toxicity we believe that this combination should be employed as induction therapy before high-dose regimens in selected patients with stage IV disease.

### REFERENCES

Antman K, Ayash L, Elias A, Wheeler C, Hunt M, Eder JP, Teicher BA, Critchlow J, Bibbo J, Schnipper LE and Frei III E (1992) A phase II study of high-dose cyclophosphamide, thiotepa and carboplatin with autologous marrow support in women with measurable advanced breast cancer responding to standard-dose chemotherapy. *J Clin Oncol* **10**: 102–110

Bender JG, To LB, Williams S, Schwartzberg LS (1992) Defining a therapeutic dose of peripheral blood stem cells. *J Hematother* **1**: 329–341

Bensinger W, Singer J, Appelbaum F, Lilleby K, Longin K, Rowley S, Clarke E, Clift R, Hansen J, Shields T, Storb R, Weaver C, Weiden P and Buckner CD (1993) Autologous transplantation with peripheral blood mononuclear cells collected after administration of recombinant granulocyte colony-stimulating factor. *Blood* **81**: 3158–3163

Bensinger W, Appelbaum F, Rowley S, Storb R, Sanders J, Lilleby K, Gooley T, Demirer T, Schiffman K, Weaver C, Clift R, Chauncey T, Klarnet J, Montgomery P, Petersdorf S, Weiden P, Witherspoon R and Buckner CD (1995) Factors that influence collection and engraftment of autologous peripheral-blood stem cells. *J Clin Oncol* **13**: 2547–2555

Bezwoda WR, Seymour L and Dansey RD (1995) High-dose chemotherapy with hematopoietic rescue as primary treatment for metastatic breast cancer: a randomized trial. *J Clin Oncol* **13**: 2483–2489

Bonadonna G, Gianni L, Santoro, Bonfante V, Bidoli P, Casali P, Demicheli R and Valagussa P (1993) Drugs ten years later: epirubicin. *Ann Oncol* **4**: 359–369

Breems DA, Van Hennik PB, Kusadasi N, Boudewijn A, Cornelissen JJ, Sonneveld P and Ploemacher RE (1996) Individual stem cell quality in leukapheresis products is related to the number of mobilized stem cells. *Blood* **87**: 5370–5378

Brugger W, Bross K, Frisch J, Dern P, Weber B, Mettelsmann R and Kanz L (1992) Mobilization of peripheral blood progenitor cells by sequential administration of Interleukin-3 and granulocyte-macrophage colony stimulating factor following polychemotherapy with Etoposide, Ifosfamide and Cisplatin. *Blood* **79**: 1193–1200

Buzdar AU, Holmes FA and Hortobagyi GN (1995) Paclitaxel in the treatment of metastatic breast cancer: MD Anderson Cancer Center experience. *Sem Oncol* **22**: 101–104

Chao NJ, Schriber JR, Grimes K, Long GD, Negrin RS, Raimondi CM, Horning SJ, Brown SL, Miller L and Blume KG (1993) Granulocyte colony-stimulating factor 'mobilized' peripheral blood progenitor cells accelerate granulocyte and platelet recovery after high-dose chemotherapy. *Blood* **81**: 2031–2035

Demirer T, Rowley S, Buckner CD, Frederick R, Appelbaum FR, Lilleby K, Storb R, Schiffman K and Besinger WI (1995) Peripheral-blood stem-cell collections after paclitaxel, cyclophosphamide and recombinant human granulocyte colony stimulating factor in patients with breast and ovarian cancer. *J Clin Oncol* **13**: 1714–1719

© Cancer Research Campaign 1997

Fennelly D, Schneider J, Spriggs D, Bengala C, Hakes T, Reich L, Barakat R, Curtin J, Moore MAS, Hoskins W, Norton L and Crown J (1995) Dose escalation of paclitaxel with high-dose cyclophosphamide, with analysis of hematologic support of advanced ovarian cancer patients receiving rapidly sequenced high-dose carboplatin/cyclophosphamide courses. *J Clin Oncol* **13**: 1160–1166

Gianni AM (1994) Where do we stand with the use of peripheral blood progenitor cells? *Ann Oncol* **5**: 781–784

Gianni AM, Siena S, Bregni M, Di Nicola M, Dodero A, Zambetti M, Salvadori B, Luini A, Greco M, Zucali R, Valagussa P and Bonadonna G (1995) 5-year results of high-dose sequential (HDS) adjuvant chemotherapy in breast cancer with > 10 positive nodes (Abstract 61). *J Clin Oncol* **13** (suppl. 1): p. 90

Gianni L, Munzone E, Capri G, Villani F, Spreafico C, Tarenzi E, Fulfaro F, Caraceni A, Martini C, Laffranchi A, Valagussa P and Bonadonna G (1995*a*) Paclitaxel in metastatic breast cancer: a trial of two doses by 3-hour infusion in patients with disease recurrence after prior therapy with anthracyclines. *J Natl Cancer Inst* **87**: 1169–1175

Gianni L, Munzone E, Capri G, Fulfaro F, Tarenzi E, Villani F, Spreafico C, Laffranchi A, Caraceni A, Martini C, Stefanelli M, Valagussa P and Bonadonna G (1995*b*) Paclitaxel by 3-hour infusion in combination with bolus doxorubicin in women with untreated metastatic breast cancer: high antitumor efficacy and cardiac effects in a dose-finding and sequence-finding study. *J Clin Oncol* **13**: 2688–2699

Holoyake TL and Franklin IM (1994) Bone marrow transplant from peripheral blood. *Br Med J* **309**: 4–5

Hortobagyi GN (1995) High-dose chemotherapy is not an established treatment for breast cancer. *Proc Am Soc Clin Oncol* Educational Book, pp. 341–346

Hortobagyi GN, Holmes FA, Theriaul RL and Buzdar AU (1994) Use of Taxol (paclitaxel) in breast cancer. *Oncology* **51** (suppl. 1): 29–32

Hows JM, Bradley BA, Marsh JCW, Luft T, Coutinho L, Testa NG and Dexter TM (1992) Growth of human umbilical-cord blood in long term hematopoietic cultures. *Lancet* **340**: 73–76

Kennedy MJ (1995) High-dose chemotherapy of breast cancer: is the question answered? *J Clin Oncol* **13**: 2477–2479

Peters WP, Shpall EJ, Jones RB, Olsen GA, Bast RC, Gockerman JP and Moore JO (1988) High-dose combination alkylating agents with bone marrow support as initial treatment for metastatic breast cancer. *J Clin Oncol* **6**: 1368–1376

Peters WP, Jones RB, Vredenburgh J, Shpall EJ, Hussein A, Elkordy M, Rubin P, Ross M and Berry D (1996) A large prospective randomized trial of high-dose combination alkylating agents (CPB) with autologous cellular support (ABMS) as consolidation for patients with metastatic breast cancer achieving complete remission after intensive doxorubicin-based induction therapy (AFM) (Abstract 149). *J Clin Oncol* **14** (suppl. 1): p. 141

Pettengell R, Luft T, Henschler R, Hows JM, Dexter TM, Ryder D and Testa NG (1994) Direct comparison by limiting dilution analysis of LTC-IC in human bone marrow, umbilical cord blood and blood stem cells. *Blood* **84**: 3653–3659

Ploemacher RE (1994) Cobblestone area forming cell (CAFC) assay. In *Culture of hematopoietic cells*, Freshney RI, Pragnell IB and Freshney MG (eds), pp. 1–21. Wiley-Liss: New York

Podda S, Ward M, Himelstein A, Richardson C, De La Flor-Weiss E, Smith L, Gottesman M, Pastan I and Bank A (1992) Transfer and expression of the human multiple drug resistance gene into live mouse. *Proc Natl Acad Sci USA* **89**: 9676–9680

Ross AA, Cooper BW, Lazarus HM, Mackay W, Moss TJ, Ciobanu N, Tallman MS, Kennedy MJ, Davidson NE, Sweet D, Winter C, Akard L, Jansen J, Copelan E, Meaghe RC, Herzig RH, Klumpp TR, Kahn DG and Warner NE (1993) Detection and viability of tumor cells in peripheral blood stem cell collections from breast cancer patients using immunocytochemical and clonogenic assay techniques. *Blood* **82**: 2605–2610

Schmitz N, Linch DC, Dreger P, Goldstone AH, Boogaerts MA, Ferrant A, Demuynck HMS, Link H, Zander A, Barge A and Borkett K (1996) Randomized trial of filgrastim-mobilized peripheral blood progenitor cell transplantation versus autologous bone-marrow transplantation in lymphoma patients. *Lancet* **347**: 353–357

Schwella N, Beyer I, Schwaner I, Heuft HG, Rick O, Huhn D, Serke S, Siegert W (1996) Impact of preleukapheresis cell counts on collection results and correlation of progenitor-cell dose with engraftment after high-dose chemotherapy in patients with germ cell cancer. *J Clin Oncol* **14**: 1114–1121

Seidman AD (1995) The emerging role of paclitaxel in breast cancer. *Clin Cancer Res* **1**: 247–256

Sheridan WP, Begley CG, Juttner CA, Szer J, To LB, Maher D, McGrath KM, Morstyn G and Fox RM (1992) Effect of peripheral blood progenitor cells mobilized by filgrastim (G-CSF) on platelet recovery after high dose chemotherapy. *Lancet* **339**: 640–644

Shimazaki C, Oku N, Ashihara E, Okawa K, Goto H, Inaba T, Ito K, Fujita N, Tsuji H, Murakami S, Harujama H, Nishio A and Nakagawa M (1992) Collection of peripheral blood stem cells mobilized by high dose ara-C plus VP16 or aclarubicin followed by rHu G-CSF. *Bone Marrow Transpl* **10**: 341–346

Shpall EJ, Jones RB and Bearman S (1994) High-dose therapy with autologous bone marrow transplantation for the treatment of solid tumors. *Curr Opin Oncol* **6**: 135–138

Siena S, Bregni M, Brando B, Ravagnani F, Bonadonna G and Gianni AM (1989) Circulation of $CD34^+$ hematopoietic stem cells in the peripheral blood of high-dose cyclophosphamide-treated patients: enhancement by intravenous recombinant human granulocyte-macrophage colony-stimulating factor. *Blood* **74**: 1905–1914

Siena S, Bregni M, Brando B, Belli N, Ravagnani F, Gandola L, Stern AK, Lansdorp PM, Bonadonna G and Gianni AM (1991) Flow cytometry for clinical estimation of circulating hematopoietic progenitors for autologous transplantation in cancer patients. *Blood* **77**: 400–409

Siena S, Bregni M, Di Nicola M, Ravagnani F, Peccatori F, Gandola L, Lombardi F, Tarella C, Bonadonna G and Gianni AM (1994) Durability of hematopoiesis following autografting with peripheral blood hematopoietic progenitors. *Ann Oncol* **5**: 935–941

Sledge GW Jr, Robert N, Sparano JA, Cogleigh M, Goldstein LJ, Neuberg D, Rowinsky E, Baughman C and McCaskill-Steens W (1995) Eastern Cooperative Oncology group studies of paclitaxel and doxorubicin in advanced breast cancer. *Semin Oncol* **22** (suppl. 6): 105–108

Torretta L, Perotti C, Dornini G, Danova M, Locatelli F, Pedrazzoli P, Preti P, Da Prada GA, Pavesi L, Robustelli Della Cuna G and Salvaneschi L (1996) Circulating progenitor cell collection: experience from 275 leukaphereses in various malignancies and healthy donors. *Haematologica* **81**: 208–215

Tricot G, Jagannath S, Vesole D, Nelson J, Tindle S, Miller L, Cheson B, Crowley J and Barlogie B (1995) Peripheral blood stem cell transplants for multiple myeloma: identification of favorable variables for rapid engraftment in 225 patients. *Blood* **85**: 588–596

WHO handbook of reporting results of cancer treatment (1979) World Health Organization: Geneva

Zimmerman TM, Lee WJ, Bender JG, Mick R and Williams SF (1995) Quantitative CD34 analysis may be used to guide peripheral blood stem cell harvests. *Bone Marrow Transplant* **80**: 439–444

© Cancer Research Campaign 1997